# Sex differences in social connectedness, health, and quality of life: evidence from a cross-sectional survey in urban Accra, Ghana

**DOI:** 10.1186/s12889-025-25322-3

**Published:** 2025-11-29

**Authors:** Rolando Leiva-Granados, Carlos Salvador Grijalva-Eternod, Irene Akwo Kretchy, Samuel Amon, Leonard Baatiema, Hassan Haghparast-Bidgoli, Akanksha A. Marphatia, Emeline Rougeaux, Mawuli Komla Kushitor, Sandra Boatemaa Kushitor, Raphael Baffour Awuah, Edward Fottrell

**Affiliations:** 1 Institute for Global Health, University College London, 3rd floor, 30 Guilford Street, London, WC1N 1EH UK; 2 London School of Hygiene & Tropical Medicine, London, UK; 3 Department of Pharmacy Practice and Clinical Pharmacy, School of Pharmacy, University of Ghana, Accra, Ghana; 4 Noguchi Memorial Institute for Medical Research, University of Ghana, Accra, Ghana; 5 Department of Health Policy, Planning and Management, School of Public Health, University of Ghana, Legon, Accra Ghana; 6 Department of Global Health and Population, Harvard T.H. Chan School of Public Health, Boston, USA; 7 Department of Health Policy, Planning and Management, Fred N. Binka School of Public Health, University of Health and Allied Sciences, Ho, Volta Region Ghana; 8 Department of Community Health, Ensign Global College, Kpong, Ghana; 9 Department of Food Science and Centre for Sustainability Transitions, Stellenbosch University, Stellenbosch, South Africa; 10 Regional Institute for Population Studies, University of Ghana, Accra, Ghana; 11 Vital Strategies, New York City, USA

**Keywords:** Quality of life, Diabetes, Self-rated health, Overweight, Obesity, Social connectedness, Social networks

## Abstract

**Background:**

Social relationships are recognised as a determinant of health and well-being. However, cultural norms, including those related to sex roles, shape how individuals form and experience social connections, and may influence how they affect health and quality of life (QoL). Yet, most existing evidence on the links between social connectedness and well-being comes from high-income countries and focuses primarily on subjective outcomes. Less is known about these associations in low- and middle-income settings, particularly when considering both objective and subjective health and QoL measures. This study explored how indicators of social connectedness were associated with health and QoL outcomes among women and men in a low-income urban neighbourhood in Accra, Ghana.

**Methods:**

We used data from the Contextual Awareness Response and Evaluation: Diabetes in Ghana project (CARE) community-based survey conducted in Ga Mashie, Ghana. We employed logistic and linear regression models to study associations between four indicators of social connectedness and a range of well-being and health outcomes, including QoL, self-rated health, diabetes risk, and overweight and obesity.

**Results:**

Women reported, on average, lower levels of social connectedness compared to men. Moreover, most statistically significant associations were found for subjective rather than objective outcomes, and these associations varied by sex. Among men, participation in associations was positively linked to psychological and environmental dimensions of QoL. For women, group participation was associated with better self-rated health. A small association also suggested that friendships could be linked to a potential negative impact on some dimensions of women’s QoL.

**Conclusion:**

Our study underscores the importance of examining sex-specific patterns in the relationships between social connectedness, QoL and health outcomes. Social connections appear to have both beneficial and non-beneficial effects, especially among women, although the magnitude of these effects was often small. This may reflect complex social dynamics shaped by cultural and traditional roles that differ by sex. Further research is needed to better understand these findings. This includes identifying potential mediating variables that explain the associations between social connectedness and individual health and QoL, and exploring why some of these associations differ by sex.

**Supplementary Information:**

The online version contains supplementary material available at 10.1186/s12889-025-25322-3.

## Background

Health and well-being are global priorities, but the specific challenges and determinants vary widely across low-, middle-, and high-income countries. While global life expectancy has improved [1], many low- and middle-income countries (LMICs) are facing a growing burden of non-communicable diseases (NCDs), alongside persistent challenges related to infectious diseases and achieving universal health coverage [2–4]. These health burdens also impact broader measures of well-being, including quality of life (QoL), a multidimensional concept referring to how individuals perceive their overall well-being and place in life, taking into account the cultural context, value systems, personal goals, expectations, and concerns that shape their daily experience [5]. A growing body of research has examined the factors influencing health and QoL, highlighting the importance of factors such as income, education, and access to healthcare [6–9], which tend to be more unequally distributed and constrained in LMIC settings.

Over the past few decades, social connectedness has gained increasing attention as a key social determinant of health and well-being [10]. In this paper, we use the umbrella term *social connectedness* to describe those social interactions, defined as *“a combination of interrelated constructs spanning social support*,* social networks*,* and the absence of perceived isolation”* [10, p. 1]. The existing literature has studied numerous indicators of social connectedness, including both the quality and quantity of friendships [11–13], social capital [14], partnerships and living arrangements with family members [15–18], and participation in associations and groups [14, 19].

**Fig. 1:**
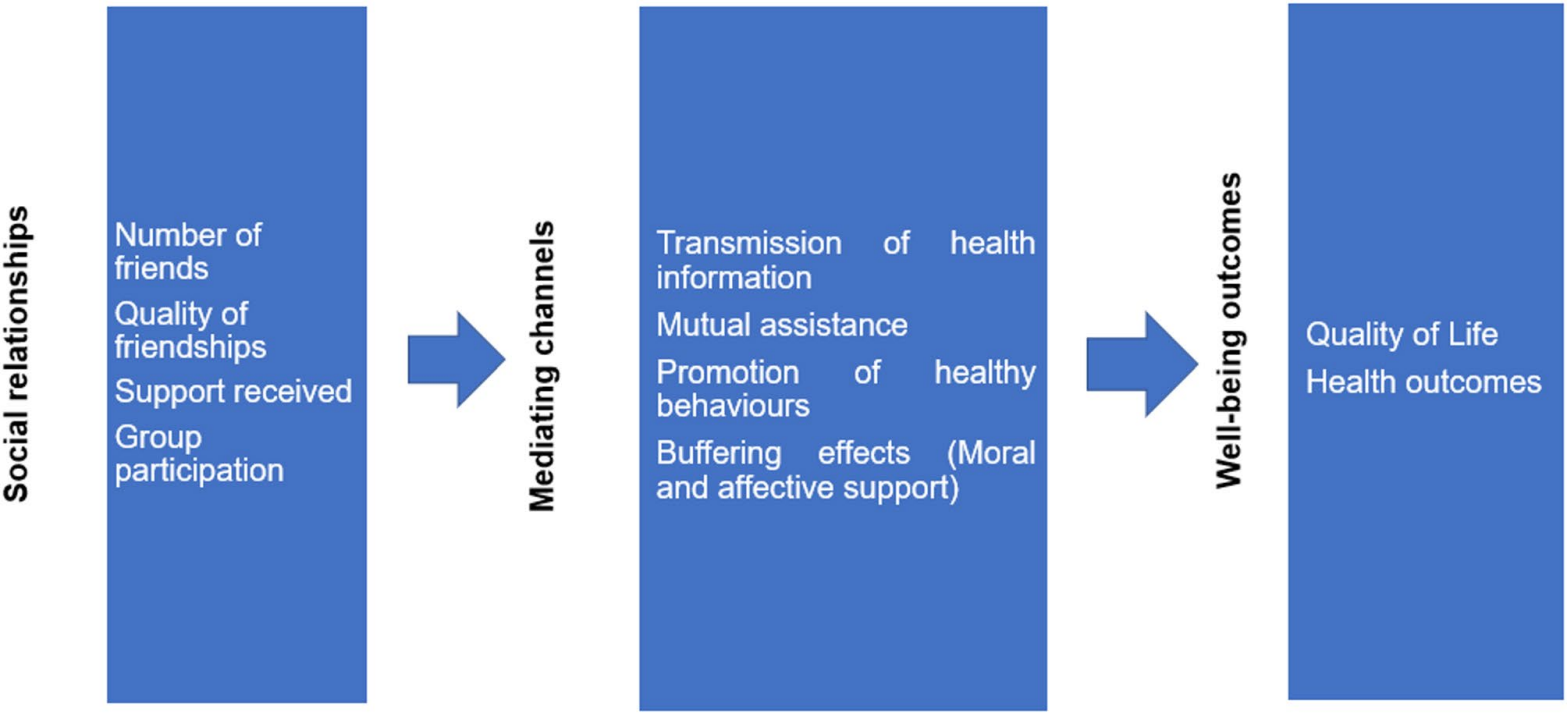
Conceptual Map: Pathway from Social Connectedness to Health and Well-Being Outcomes. Source: adapted from Fiorilli and Sabatini [11]

An overview of a potential pathway from social connectedness to an individual’s well-being is shown in Fig. 1 below. As discussed by Fiorillo and Sabatini [11], social interactions may improve health and QoL through four key mechanisms: the transmission of health information, mutual assistance, the promotion of health behaviours (also called peer effects), and buffering effects. The latter refers to the moral and affective support that can aid individuals in navigating periods of illness or, alternatively, prevent mental health issues, including depression, among those who are otherwise healthy.

While social connections often benefit health, they can also have negative effects. Some studies have linked peer influence to behaviours like smoking, alcohol use, and weight gain [20–23]. For example, Christakis et al. found that the likelihood of becoming obese increased by 57% if a close friend became obese, by 40% if a sibling did, and by 37% if it was a spouse [22]. Moreover, a literature review reported that individuals with similar body weights were more likely to share network ties, and that the characteristics of friendship networks influence changes in body weight [23].

Norms associated to sex and gender are factors shaping social interactions. Men often engage in structured networks through work or associations, while women are more likely to form close and supportive relationships with family and friends [24, 25]. Women also provide more frequent and intensive care to their peers, which may increase their exposure to others’ stress and problems and negatively affect their own health [18, 25, 26]. This highlights the importance of analysing social connectedness by sex.

In recent decades, health interventions have increasingly drawn on people’s social environments. Community-based programmes, peer-led initiatives, and participatory approaches that engage existing networks have gained prominence [27, 28], and are often seen as effective strategies for improving population health.

However, literature reviews and meta-analyses have raised questions about the role of social connectedness in shaping health and well-being. For example, the meta-analysis by Xue et al. [29] found only a small association between social relationships and health, along with evidence of positive publication bias, though its impact on average effect sizes was limited. Another review [14] reported that associations between social capital and health were more commonly observed in studies using self-rated health outcomes, with fewer studies examining objective health indicators. Finally, evidence on the effectiveness of interventions based on social connectedness to improve health and QoL remains mixed and even contradictory [30, 31].

While these debates continue, they are still largely informed by evidence from high-income countries (HICs). For example, a review by Schram et al. [32] found that only one of 17 studies on social networks and diabetes included participants from LMICs. Similarly, a systematic review of longitudinal studies on social ties and obesity identified only studies from the United States [33].

However, social interactions are highly context dependent. In LMICs, social networks and family structures often differ from those in HICs [34]. As previously discussed, LMICs also face a disproportionate burden of disease and disability [3, 35], and weaker health and social care systems may lead people to depend more on family and community support. These contextual differences limit the direct applicability of findings from HICs to LMICs settings [34].

Therefore, given the ongoing debate about the effects of social connectedness on health and QoL, including the less frequent use of objective outcomes, the existence of positive publication bias and overreliance on results from HICs settings, it is essential to continue studying the effects of social connectedness on health and QoL in LMIC settings.

This study explored how indicators of social connectedness were associated with subjective and objective health and QoL outcomes among women and men in an urban poor neighbourhood in Ga Mashie, Accra, Ghana.

Ga Mashie offers a valuable setting to examine the links between social relationships, health and QoL in LMICs, particularly in sub-Saharan Africa. Ghana and the wider sub-Saharan region are undergoing rapid and unplanned urbanisation [36], which has changed social ties and created new health challenges, including an increase in non-communicable diseases such as diabetes and obesity [37]. Moreover, in urban Accra, gaps in public healthcare often force residents to turn to private providers [38]. Additionally, Ghanaian society is strongly collectivist [39], placing great value on family and community life, while gender norms contribute to inequalities in access to economic resources [40].

In this context of high disease burden, healthcare provision gaps and persistent sex roles, social connections may be key in shaping health and QoL. Studying these dynamics can help clarify the strength of these associations, test for possible null effects, examine sex differences, and inform policy aimed at improving health and well-being in Ghana and similar LMICs.

## Methods

### Setting and design

This study used data from the Contextual Awareness, Response and Evaluation (CARE) Diabetes Project in Ga Mashie, Accra, Ghana. CARE is an interdisciplinary initiative aimed at understanding diabetes in urban poor communities in Ghana. It involves multiple components, including qualitative and quantitative data collection, data analysis and stakeholder engagement. This paper draws on data from a household survey conducted in the framework of this project. Ga Mashie, which includes the neighbourhoods of James Town and Ussher Town, is a densely populated area which spans approximately 100 hectares with an estimated 120,000 residents. It is marked by widespread poverty, low levels of literacy, inadequate sanitation, and largely aging housing infrastructure [41].

A comprehensive protocol for the household survey has been published elsewhere [41]. In summary, a random sample survey was conducted between November 10th and December 8th, 2022. The survey focused on Ga Mashie residents aged 25 and above, as NCD risk factors and health system interactions increase significantly after early adulthood, making this age group most relevant for policy, planning, and efficient resource use.

Participants were recruited from 959 randomly selected households across 80 enumeration areas (clusters) identified in the 2021 Population and Housing Census. Each household was visited by trained enumerators, and the data were collected following written informed consent. The questionnaire was administered in Ga, the local language.

Although the main aim of the project was to examine the prevalence of diabetes and NCD risk factors among adults in the community, it also gathered extensive data on social determinants of health (including social connectedness), health status, QoL and demographic and socioeconomic characteristics of participants. Objective measures of random blood glucose, weight, height, and blood pressure were taken using standard methods. To enhance the accuracy of blood glucose and blood pressure measurements, pregnant women or those who had given birth within the last six months were excluded from the study.

### Sample flow

A detailed description of the sample characteristics is provided by Grijalva-Eternod et al. [42]. In summary, from the 959 households visited, 67% of households (*n* = 644) agreed to participate in the survey (95% CI: [61%−72%), while 32% (95% CI: [26.6%−37.5%) (*n* = 306) were not found and 1% (95% CI: [0.83%−3.11%) (*n* = 9) refused to participate. Of the 1,007 eligible individuals from the 644 households that agreed to participate in the study, 13.5% (*n* = 135) were absent and 1.8% (*n* = 18) did not consent to be surveyed. As a result, 854 individuals from 629 households were surveyed.

Previous analysis of the sample [42] showed that non-response (due to absence or refusal) was more common among younger individuals (20.4% in ages 25–44 vs. 3.7% among those aged ≥ 65), men (19.9% vs. 12.3% in women), and those in higher wealth tertiles (16.8% in the least poor vs. 12.1% in the most poor). However, these patterns of non-participation did not substantially alter the age, sex, or wealth distribution of the surveyed sample compared to the eligible population, suggesting that the sample remained broadly representative.

Data from a final working sample of 839 individuals were used for the statistical analysis involving association membership and one-person households as main regressors (see next section). The reduction in the sample size from 854 to 839 was due to 15 individuals having missing information for at least one of the confounding variables.

### Variables

This section summarises the outcome variables, indicators of social connectedness, and the covariates used to adjust for potential confounding.

#### Outcome variables

The primary outcome variables for this study were the following subjective and objective measures of QoL and health status:

##### Quality of life measures

As described in the background section, QoL is a subjective measure of individuals’ perception of their position in life, which is shaped by their cultural, social, and environmental context [5]. QoL measures are widely used in both population health and clinical settings, as well as in research on social connectedness [43–45].

In our survey, we assessed QoL using the World Health Organization Quality of Life (WHOQOL-BREF) questionnaire [5]. The self-reported general QoL indicator was derived from the response to the question *‘How would you rate your quality of life?*’ with five categories: very poor, poor, neither poor nor good, good, very good. To make the results easier to interpret, we dichotomised the QoL indicator into two categories, where 1 indicated good or very good QoL, and 0 otherwise. This approach is in line with previous studies that used similar methods to analyse ordered variables with logistic regression [46, 47]. However, in Tables A4 and A5 (see Additional File 3, tables for the Online Appendix), we include the results from an ordered logistic regression using the original five-point scale, which show that the overall findings are similar to those using the binary variable.

It is well recognised that life satisfaction, measured here using the QoL indicator, is a multidimensional concept [5]. The WHOQOL-BREF questionnaire includes four dimensions of QoL: physical health, psychological well-being, social relationships, and environment.

Data were collected for each of these four dimensions using Likert scales. The physical dimension of QoL included, for example, information on pain and discomfort, mobility, and work capacity, while the psychological dimension covered aspects such as positive and negative feelings and self-esteem. The social relationships dimension included items on personal relationships and social support, while the environmental dimension covered elements such as the home environment, freedom, physical safety and security. A full list of the specific information that the WHOQOL-BREF questionnaire collects in each of the four domains is available in [5]. Following [48], an index for each dimension was constructed on a scale from 0 to 100, with higher scores indicating better QoL.

##### Health measures

We used a combination of subjective and objective measures of health status. Subjective health was assessed through self-rated health, where individuals were requested to rate, on a scale ranging from 0 to 10, the perceived quality of their health on the day of the survey. Higher values on this scale indicate a more positive perception of health [49].

As objective health outcomes, our study included the risk of diabetes and overweight (including obesity), both recognised as key risk factors for cardiovascular disease [50, 51], which remains one of the major causes of death in Ghana and sub-Saharan Africa [37, 52].

Diabetes risk was assessed through a dual criterion: individuals reporting a previous diabetes diagnosis or those with a random blood glucose concentration of 11.1 mmol/L or higher at the time of the survey. Individuals meeting either criterion were coded as 1 for diabetes risk, and 0 otherwise. Random blood glucose levels were determined using a point-of-care glucometer, with capillary blood obtained from the middle finger.

The variable for overweight and obesity was derived from BMI measurements obtained during the survey. It was constructed as a binary indicator, taking the value of 1 if an individual’s BMI was equal to or exceeded 25, and 0 otherwise. Robustness checks were carried out using a variable representing obesity only, coded as 1 for BMI ≥ 30 and 0 otherwise. The results (presented in Tables A4 and A5, see Additional File 3 - tables for the Online Appendix) did not differ from those obtained using the combined overweight and obesity variable.

#### Indicators of social connectedness

Social connectedness is an umbrella term for many different types of social interactions, including broader concepts such as “social networks” or “social capital”. It is therefore not surprising that previous studies have used a wide range of variables to measure these [10], along with diverse tools for collecting such information in the context of household or individual surveys.

However, the literature has noted that many of the instruments used to collect this information have been developed in HICs, and thus requiring cultural adaptation of the proposed measures when applied in LMICS [53]. This is particularly important considering that social structures, such as the family, often have different configurations and functions in LMICs compared with HICs settings [34].

In this study, we use four simple indicators of social connectedness: participation in community associations, living in a one-person household, the number of close friends, and the absence of friends (reporting zero close friends). All of these measures are shaped by the specific cultural context of a sub-Saharan African country like Ghana, particularly given that, with the exception of living in a one-person household, they are subjective in nature.

Participation in community associations is represented by a binary variable, determined by asking participants if they were members of any association or group in the community. This was coded as 1 for participation and 0 otherwise. As shown in Table A1 (see Additional File 3, tables for the Online Appendix), the most common group of attendance was religious associations (49%), a point further considered in the discussion section in light of our results.

Living in a one-person household is a binary variable, taking the value of 1 if an individual resided without any other relatives in the same house. A value of 0 denoted the case were the individual shared a home with members of the same household. In studies conducted in HICs, similar variables such as “living alone” have been used [16, 17]. However, it is important to note that, in the context of urban Accra’s familial arrangements, even if an individual qualifies as a one-person household, they may share the same dwelling with people from other households. For this reason, we refer to the variable as “living in a one-person household” rather than “living alone”.

The number of close friends is a discrete variable, originally based on responses ranging from 0 to 100. Information for this variable was obtained through the following question: *“How many friends would you say you have close relationships with in this community?”.* To focus on more realistic values, we capped the variable at two different maximums: 10 and 20 friends.

We chose these limits based on the data distribution. The 75th percentile for this variable was 10 friends, and, as shown in Additional File 2 (Figure A1 for the Online Appendix), the upper adjacent value (75th percentile + 1.5 × interquartile range) was 20 friends. Responses above 20 were treated as outliers and removed from the analysis. After this adjustment, the sample size was reduced from 839 to 760 for the 0–20 range, and to 686 for the 0–10 range. The main analysis used the variable capped at 10. Results using the 0–20 version were largely consistent with those findings, with any differences discussed in the Results Section.

To capture cases of social isolation, we dichotomised the variable of number of friends, assigning a value of 1 to individuals who reported having no close friends and 0 for those who reported at least one. As with the discrete version, we conducted robustness checks for this variable using the original number of friends variable capped at 20.

Finally, regarding multiple indicators of lack of social connectedness, only two people reported simultaneously having zero friends, living in one-person households, and not participating in associations. Additionally, only 32 people reported experiencing a combination of two out of these three indicators. Among those who reported not having friends, 11 lived in an one-person household and 17 participated in associations. Due to these small sample sizes, no estimations based on these combined measures were conducted.

#### Adjustment of confounding variables

To control for potential confounders, we included the following variables in the regression analyses: age and its squared term (to account for non-linear effects of age), marital status (married or living together, divorced/separated, widowed and never married), education level (no education, pre-school completed, primary completed, middle/Junior High School (JHS) completed, secondary/Senior High School (SHS) completed and higher education completed), employment status (currently working or not), frequency of religious attendance (less than once a month or never, a few times a month, at least once a week, and nearly every day), and a wealth index divided in quintiles. The wealth index was constructed using principal component analysis based on household assets, such as access to a television, washing machine, main water sources, and number of rooms.

### Regression analyses

We used multivariate regression analysis to study the associations of our variables of social connectedness with health and QoL, adjusting for the relevant covariates described above. For the dichotomous variables of QoL, overweight and risk of diabetes, we used logistic regression and report odds ratios (OR) with 95% confidence intervals (CI). For the continuous indexes of self-rated health and QoL subdimensions, we report the coefficients from linear regression models and their 95% CI. To facilitate interpretation of the magnitude and practical significance of the linear regression findings, we calculated relative effect sizes. These were derived by dividing the regression coefficient, which represents the marginal change in the outcome, by the average value of that outcome and multiplying by 100. This approach was applied only to linear regression coefficients, as effect sizes from odds ratios are generally more straightforward to interpret.

Separate models were estimated for each of the four measures of social connectedness. Analyses were also conducted separately by sex, with results for the entire sample presented in Tables A2 and A3 (see Additional File 3, tables for the Online Appendix).

All estimations were performed in Stata 18.5, incorporating the survey design by adjusting our estimates using probability weights and clustered standard errors at the enumeration area level. Clustering the standard errors makes them robust to heteroskedasticity and relaxes the assumptions of normally distributed errors, as well as the assumption of independently and identically distributed errors [54, 55].

We assessed multicollinearity using the Variance Inflation Factor (VIF) through Stata’s “vif” postestimation command. All variables had VIF values below the recommended threshold of 10, indicating no significant multicollinearity concerns. The only exception was the variables age and age squared, which are expected to be correlated. Their inclusion was based on previous literature that has found non-linear relationships between age and health outcomes [56]. This correlation is therefore not considered problematic and does not compromise the validity of the model.

We adjusted for the rich set of variables described above to control for potential confounding. Nonetheless, given the cross-sectional nature of our study, there remains the possibility of residual confounding. This is acknowledged as a potential limitation of our study.

## Results

### Descriptive statistics

Table 1 presents the descriptive statistics for the study participants. The sample comprised more women than men (64% vs. 36%). The mean age of the participants was 48 years old, with 73% being in employment at the time of the survey. Almost half of the study participants were either married or cohabiting with their partner, while 18% had never been married. Most of the sample (40%) completed middle school, 11% reported no education, and only 6% had attained higher education.

**Table 1:** Selected descriptive statistics for the sample

Variables	*N*	Weighted percentage [95% confidence intervals]
Female sex	839	64% [60%,67%]
Currently working	839	73% [69%,77%]
Age in years	839	48 [47, 50]
Marital status		
*Currently married or living together*	403	49% [44%, 53%]
*Divorced/Separated*	155	18% [15%, 22%]
*Widowed*	130	15% [13%, 18%]
*Never married*	151	18% [15%, 22%]
*Total*	839	
Education		
*No education*	82	11% [8%, 14%]
*Pre-school*	16	2% [1%, 4%]
*Primary*	173	19% [16%, 23%]
*Middle/JHS*	336	40% [37%, 44%]
*Secondary/SHS*	181	22% [19%, 25%]
*Higher*	51	6% [4%, 10%]
*Total*	839	

Table 2 presents descriptive statistics for the outcome variables and indicators of social connectedness, disaggregated by sex.

**Table 2:** Outcomes and indicators of social connectedness by sex

Variable	Men (weighted percentage or mean and 95% CI)	Women (weighted percentage or mean and 95% CI)	Difference and *p*-values for test for statistical significance ^a/^
Outcomes			
Good or very good QoL ^b/^, percentage	57%	52%	5%
	[50%,65%]	[47%, 58%]	
Diabetes risk, percentage	4%	11%	−7% ***
	[2%, 7%]	[8%,14%]	
Overweight and obesity, percentage	45%	74%	−29% ***
	[37%,52%]	[70%,78%]	
Self-rated health, mean index	5.6	5.1	0.5 *
	[5, 6]	[4.5,5.7]	
QoL: physical health dimension, mean index	75	71	4 ***
	[73,77]	[69,72]	
QoL: psychological dimension, mean index	71	70	1
	[69,73]	[68,72]	
QoL: social dimension, mean index	68	66	2
	[66,70]	[64,69]	
QoL: environment dimension, mean index	58	57	1
	[56,60]	[55,59]	
Indicators of social connectedness			
Belonging to an association or group, percentage	23%	14%	9% ***
	[18%,30%]	[10%, 19%]	
Living in a one-person household, percentage	11%	2%	−9% ***
	[8%,16%]	[1%,4%]	
Having no close friends, percentage	16%	24%	−8% *
	[10%,24%]	[18%,30%]	
Number of close friends, mean	4	3.4	−0.6 **
	[3.5,4.6]	[3,3.7]	

a/Test for statistical significance for the difference between men and women. A t-test was used for continuous variables and a chi-square test for binary variables. *P*-vales: *** *p* < 0.01; ** *p* < 0.05; * *p* < 0.1

b/QoL: Quality of life

In terms of outcomes, women showed higher prevalence of diabetes as well as overweight and obesity compared to men. The difference in overweight and obesity was particularly striking: 74% of women were classified as overweight or obese, compared to 45% of men. Women had a lower mean value on the physical dimension of QoL, with a mean index of 71 versus 74 for men. No statistically significant differences were observed for the other outcomes.

Regarding indicators of social connectedness, men were generally in a more favourable position. A higher proportion of men reported belonging to associations (23% vs. 14%), had a greater number of close friends on average (4 vs. 3.4), and fewer men reported having no close friends (16% vs. 24%). However, a greater share of men lived in one-person households (11% vs. 2% among women).

Due to the low numbers of individuals living in one-person households, particularly women, and the lower prevalence of diabetes among men, some estimations using these variables had wide confidence intervals, indicating low statistical precision.

### Regression results

In this section, we present the associations between social connectedness and our outcome variables by sex of the respondent (see Table 3 in Additional File 1). Results for the entire sample are available in Tables A2 and A3 (see Additional File 3, tables for the Online Appendix).

First, the relationship between association belonging and self-rated health differed between women and men. While no statistically significant association was observed for men, higher association belonging was associated with a 1.3 point higher self-rated health index among women (95% CI: [0.5, 2.0]). Given that the mean self-rated health score for women was 5.1 (see Table 2), this reflects an increase of approximately 25% relative to the average score.

Moreover, living in a one-person household was associated with a high odds ratio of 5.3 for diabetes risk among men. However, given the wide confidence intervals (95% CI: [1.2, 24]), this effect lacks statistical precision. None of the women living alone had diabetes, so this model was not estimated for them.

### Regression results by dimensions of quality of life

Table 3 (Additional File 1) showed an overall lack of statistically significant results for the impact of social connectedness on the probability of reporting good or very good QoL. In Table 4 (see Additional File 1), we present a more detailed analysis by reporting the results for the four sub-indexes of the WHOQOL-BREF QoL measurement.

First, having no friends was associated with a 4.4-point increase in the physical dimension of QoL for women (95% CI: [1.4, 7.4]), while the association was not statistically significant for men. Given the average physical QoL score for women was 71, this represented an average increase of 6%.

A similar pattern was observed for the psychological dimension of QoL. Here, having no friends was associated with a 3.8-point increase (95% CI: [0.08, 7.5]) for women, while the effect for men was not statistically significant. This corresponded to an average increase of approximately 5%, based on the index mean value of 70.

Finally, among men, participation in associations or groups was linked to a 5-point increase in psychological QoL (95% CI: [0.9, 9.2]) and a 3.3-point increase in environmental QoL (95% CI: [0.4, 6.3]). These correspond to effect sizes of approximately 7% and 6% relative to the mean scores of 71 and 58, respectively. No statistically significant effects were observed for women.

### Robustness checks

As described in the methods section, alternative models and specifications were tested. The results for these robustness checks are available on Tables A4 and A5 (see Additional File 3, tables for the Online Appendix). First, the models using the obesity variable (coded as 1 if BMI ≥ 30, 0 otherwise) yielded similar results to those using the “overweight and obesity” variable; that is, none of the associations were statistically significant. Similarly, all associations in the estimations using the original 5-point QoL scale, estimated using an ordered logit model, were not statistically significant. This is consistent with the results from the binary QoL variable.

Small differences were observed for the variables on number of friends and having no friends when using the version capped at a maximum of 20 friends, compared to the versions of these variables with a maximum of 10 friends (see Sect. 2.3.2). Firstly, number of friends (capped at 20 friends) was also positively associated with the social relationships dimension of QoL (coefficient = 0.3, 95% CI: [0.1,0.6]) and the environment dimension of QoL (coefficient = 0.4, 95% CI: [0.1,0.7]). These estimated coefficients were similar in magnitude to those obtained using the 10-friends threshold, with the main difference being the statistical significance. However, all the effect sizes were small: approximately a 4% increase for the binary QoL variable, and less than 1% increase for the social relationships and environment sub-indices.

Moreover, as in the results using the variable derived from the number of friends capped at 10, we observed a positive association between reporting no friends and the physical health dimension of QoL using the alternative version capped at 20. In this case, the estimated coefficient was 4 (95% CI: [1, 7]), very close to the coefficient of 4.4 in the estimation using the original variable. In contrast, with the alternative variable, the relationship between having no friends and the psychological dimension of QoL was not statistically significant.

## Discussion

### Summary of findings

In this paper, we assessed the association between social connectedness and objective and subjective well-being in an urban population in Accra, Ghana. We began by documenting some raw differences in QoL, our health measures and our indicators of social connectedness based on the respondent’s sex. These differences showed that women were at a relative disadvantage compared to men. Consequently, further regression analyses were performed to explore potential heterogeneous effects by sex and outcomes.

The regression analyses yielded four main findings. First, many of the estimated associations were not statistically significant, particularly those between social connectedness and objective health measures such as diabetes risk and overweight or obesity. Second, when statistically significant associations were observed, they were mainly found in subjective outcomes such as self-rated health and the different QoL dimensions, and these varied by sex. Third, most of the significant associations were small in magnitude, suggesting that while social connectedness may influence perceived health and well-being, its overall impact could be limited or shaped by contextual and gender-specific factors. Finally, these relationships were complex, as social connectedness was linked to both positive outcomes and certain unexpected negative associations.

### Objective vs. subjective measures of health and QoL

Our finding that most associations were not statistically significant appears to contrast with previous evidence reporting significant effects of social connectedness variables on health conditions such as diabetes and obesity [16, 22, 32]. However, as noted in the literature review by Rodgers et al. [14], most associations between indicators of social connectedness and health have been observed for subjective rather than objective health measures. Our results align with this pattern: aside from the association between living alone and diabetes risk among men, statistically significant associations were found only for subjective indicators of health and QoL.

This is also in line with two previous studies from Ghana. A recent hospital-based study found that neither social support nor social network characteristics were associated with diabetes control among patients with diabetes [15]. In contrast, another study focusing on subjective health and well-being reported a positive association between these outcomes and indicators of social participation [57]. However, neither of these studies examined objective and subjective indicators simultaneously, as is done in the present analysis.

Several factors may account for this pattern. First, social connectedness may not offer sufficient protection against the structural drivers of poor health in deprived communities like Ga-Mashie. These include limited access to healthcare, inadequate water and sanitation infrastructure, poor diet quality, and chronic poverty, factors identified as key barriers to improving health outcomes in urban Ghana and across sub-Saharan Africa [38, 58].

Second, as depicted in our conceptual map, any effect of social connectedness on objective health is likely to be mediated by health-related behaviours such as improved diet, physical exercise, or regular preventive health care use [11]. These behaviours are not captured in our data, and their effects on health may take time to manifest. In contrast, subjective health and QoL outcomes are more likely to be influenced in the short term by emotional support, a sense of belonging, and the frequency and quality of daily social interactions, as well as by emotional burdens or social pressures. In line with this, in this study (and in many others), most indicators of social connectedness are based on subjective perceptions. This overlap between how social connectedness is measured and the nature of the outcomes likely contributes to the stronger associations observed with subjective health in both our findings and the broader literature.

### Small effect sizes and sex differences

Sex differences also emerged in the associations between social connectedness, health and QoL. However, these findings should be interpreted with caution. The strong association between living alone and diabetes risk among men had wide confidence intervals, indicating limited statistical precision. Beyond this, the only association with a substantial effect size was that between women’s participation in associations and self-rated health, the marginal effect corresponding to an approximate 25% increase in this indicator. The remaining associations had effect sizes below 7%.

These small effect sizes are not surprising in light of the literature. Although many studies have reported associations between indicators of social connectedness and various health outcomes (and these findings been used to inform policy recommendations) the meta-analysis by Xue et al. found that such associations were consistently small across a range of health outcomes [29].

Differences in the types of groups that women and men participate in may help explain the observed sex-specific associations between group membership and health and QoL. As shown in Table A1 (see Additional File 3, tables for the Online Appendix), women were more likely to participate in religious associations than men (63% versus 34%). Additionally, while overall participation in recreational and sports groups was low (16% for the entire the sample) only 4% of women reported participation in these groups compared to 28% of men.

Existing literature has shown that religious participation and engagement in religious groups have been found to have some direct and indirect positive effects on self-rated health, both in Ghana [57] and in European countries [59]. This helps to explain our findings on women’s self-rated health and group participation.

We also documented that the psychological dimension of QoL for men was positively associated with participation in groups or associations. Previous evidence indicates that organisations where men can socialise and engage in community-based initiatives, such as Men’s Sheds in the UK or Australia, can enhance social networks and have a positive impact on men’s health and well-being outcomes [60, 61]. Consequently, initiatives aimed at improving men’s social networks and increasing awareness of the importance of providing social and psychological support to peers may enhance the benefits men derive from their connections with friends. However, for these to be effective, they need to be adapted to the specific contextual factors in Ghana and other LMICs.

In addition, men’s environmental dimension of QoL was positively associated with group association, while the effect for women was non significant. As noted earlier, women’s participation in sport groups was lower compared with men. Women in urban Ghana and other LMICs face cultural and structural barriers for sports participation, such as gender biases and limited access to sports clubs and safe public spaces [62, 63], which can negatively impact health and QoL outcomes. Therefore, initiatives to improve access to public and safe recreational spaces may increase participation in sports and physical exercise among women [64], potentially enhancing their health and QoL.

Finally, we found that reporting no close friends could be potentially associated with better physical and psychological QoL among women. This was an unexpected and somewhat counterintuitive finding. However, it is important to note that these effects were small in magnitude and that, in robustness checks using the alternative variable for having no friends, the result was statistically significant only for the physical dimension of QoL. It is also important to consider that reporting no close friends does not necessarily imply the absence of acquaintances or access to other forms of social support.

This result could, nevertheless, suggests also the presence of complex social dynamics that may influence women’s well-being, as suggested by previous literature. Research highlights that women and men derive different benefits from social relationships [24–26], and that the nature of these relationships often varies by sex. Women are more likely to maintain close ties with relatives or friends who provide emotional support and act as confidants [24, 25]. However, these same supportive relationships can also involve regular exposure to others’ stress and difficulties, which may, in turn, negatively affect women’s own mental well-being [25, 26]. In a collectivist society with strong gender norms like Ghana [39, 40], such dynamics may be intensified. This could help explain why reporting no close friends was unexpectedly associated with improved outcomes in some dimensions of QoL for women.

### Strengths and limitations

The main strength of this paper lies in the richness and diversity of the data collected in the CARE project. In comparison to some of the previous works that were limited to studying either subjective or objective measures of well-being, our study includes a range of subjective and objective measures of QoL and health outcomes, as well as four dimensions of social connectedness.

Nevertheless, this paper has four potential limitations. First, the cross-sectional nature of the data impedes obtaining causal results. Our estimated models control for a rich set of relevant confounders to reduce potential biases. However, given the unavailability of variables suitable for an instrumental variable technique, we cannot rule out that the associations observed in this study may suffer from some degree of bias due to remaining unmeasured confounding.

A second limitation is the nature of the diabetes variable. The diabetes risk variable was defined as those individuals reporting a previous diabetes diagnosis or those exhibiting a random blood glucose concentration equal to or exceeding 11.1 mmol/L at the time of the survey. The random blood glucose measure may overestimate glucose levels if the individual has had a recent meal before the survey. For this reason, as explained before, we refer to it in this study as diabetes risk, rather than diabetes.

Third, multiple models were estimated and that only a subset of associations reached statistical significance. However, all model estimates, including those that were not statistically significant, are presented either in the main text or in the online appendix. Moreover, key variables that did not achieve statistical significance, such as those related to objective health outcomes, are explicitly discussed in the paper.

Finally, while some differences in non-response rates were observed across age, gender and wealth groups, the final sample remained broadly representative of the eligible population in terms of these characteristics (see Methods Section and [42]). Nevertheless, the potential for non-response bias should be considered when interpreting our results.

## Conclusions

In this paper, we examined the effect of various indicators of social connectedness on subjective and objective measures of health and quality of life (QoL) in an urban community in Ghana. We found that social connectedness was primarily associated with subjective outcomes, and that these associations varied by sex. Furthermore, with the exception of women’s self-rated health, the observed associations were of small magnitude, which is consistent with results from previous literature [29].

These findings suggest that while social connectedness plays a role in perceived health and QoL, its overall impact may be limited or heavily influenced by broader contextual and gender-specific factors. Policy interventions aimed at enhancing social connectedness should therefore be targeted and context-sensitive. For example, enhancing men’s participation in social groups or community initiatives may improve their psychological well-being. For women, addressing cultural and structural barriers to sports and recreational participation by improving access to safe public spaces could contribute to their health and QoL.

However, given the pattern of small effect sizes found in this and other studies, these types of interventions alone may not be sufficient to significantly improve population health and QoL. These recommendations should be considered alongside broader efforts to address structural barriers such as healthcare access, sanitation and poverty.

Finally, further research is needed to better understand these findings, including identifying potential intermediate variables explaining the associations between social connectedness, health and QoL, and exploring why some of these associations differ by sex.

## Supplementary Information

Supplementary Material 1. Tables 3 and 4.

Supplementary Material 2. Figure A1. Box plot for the distribution of reported number of close friends.

Supplementary Material 3. Tables for online appendix.

## Data Availability

The datasets used and/or analysed during the current study are available from the corresponding author on reasonable request.

## Abbreviations

QoL: Quality of life
CARE: Contextual Awareness, Response and Evaluation - Diabetes in Ghana Project
WHOQOL-BREF: World Health Organization Quality of Life questionnaire
OR: Odds ratios
CI: Confidence intervals
