# Supplementary figures and images for: Sex differences in social connectedness, health, and quality of life: evidence from a cross-sectional survey in urban Accra, Ghana
Source: BMC Public Health. 2025 Nov 29;26:45. doi: 10.1186/s12889-025-25322-3 (PMC12771860; doi:10.1186/s12889-025-25322-3)

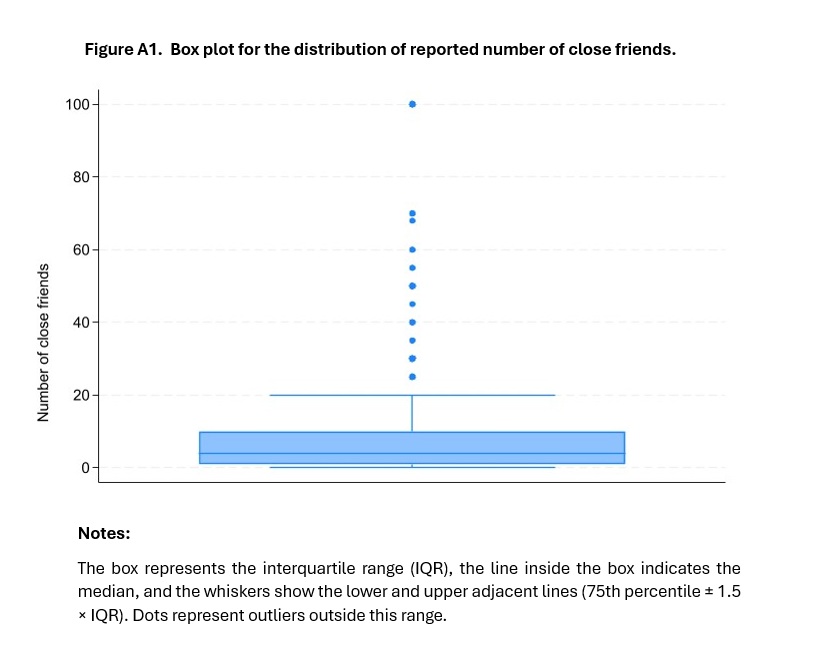

Supplement: Supplementary file 2 — Supplementary Material 2. Figure A1. Box plot for the distribution of reported number of close friends. [file 12889_2025_25322_MOESM2_ESM.jpg]
